# 

**Published:** 1872-05

**Authors:** 


### Obituary.

Died, March 31st, SAMUEL HENRY DICKSON, M.D., Professor of the Theory and Practice of Medicine in Jefferson Medical College, Philadelphia. At the time of his death, Professor Dickson had reached the ripe age of seventy four. Few names are as widely known and as highly respected by the profession.

PARTICULAR NOTICE.

☞ *THE JOURNAL will not be sent, after the present number, to any who have not renewed their subscriptions.*

☞ **Remit $3 for the current volume, commencing January, 1872.**

☞ **Back Nos. for present year can be furnished.**

